# Role of fibroblast growth factor 8 (FGF8) in animal models of osteoarthritis

**DOI:** 10.1186/ar2474

**Published:** 2008-08-12

**Authors:** Masako Uchii, Tadafumi Tamura, Toshio Suda, Masakazu Kakuni, Akira Tanaka, Ichiro Miki

**Affiliations:** 1 Pharmaceutical Research Center, Kyowa Hakko Kogyo Co., Ltd, 1188 Shimotogari, Nagaizumi, Sunto, Shizuoka 411-8731, Japan; 2 Present address: PhoenixBio Co., Ltd, 3-4-1 Kagamiyama, Higashi-Hiroshima, Hiroshima 739-0046, Japan; 3 Department of Pathology, Jichi Medical University, 3311-1 Yakushiji, Shimotsuke, Tochigi 329-0498, Japan

## Abstract

**Introduction:**

Fibroblast growth factor 8 (FGF8) is isolated as an androgen-induced growth factor, and has recently been shown to contribute to limb morphogenesis. The aim of the present study was to clarify the role of FGF8 in animal models of osteoarthritis (OA).

**Methods:**

The expression of FGF8 in the partial meniscectomy model of OA in the rabbit knee was examined by immunohistochemistry. The effect of intraperitoneal administration of anti-FGF8 antibody was tested in a model of OA that employed injection of monoiodoacetic acid or FGF8 into the knee joint of rats. The effect of FGF8 was also tested using cultured chondrocytes. Rabbit articular chondrocytes were treated with FGF8 for 48 hours, and the production of matrix metalloproteinase and the degradation of sulfated glycosaminoglycan in the extracellular matrix (ECM) were measured.

**Results:**

The expression of FGF8 in hyperplastic synovial cells and fibroblasts was induced in the meniscectomized OA model, whereas little or no expression was detected in normal synovium. Injection of FGF8 into rat knee joints induced the degradation of the ECM, which was suppressed by anti-FGF8 antibody. In the monoiodoacetic acid-induced arthritis model, anti-FGF8 antibody reduced ECM release into the synovial cavity. In cultured chondrocytes, FGF8 induced the release of matrix metalloproteinase 3 and prostaglandin E_2_, and caused degradation of the ECM. The combination of FGF8 and IL-1α accelerated the degradation of the ECM. Anti-FGF8 antibody suppressed the effects of FGF8 on the cells.

**Conclusion:**

FGF8 is produced by injured synovium and enhances the production of protease and prostaglandin E_2 _from inflamed synoviocytes. Degradation of the ECM is enhanced by FGF8. FGF8 may therefore participate in the degradation of cartilage and exacerbation of osteoarthritis.

## Introduction

Osteoarthritis (OA) is a degenerative disease and a major cause of disability in humans. Aging, mechanical stress and traumatic injury, genetic susceptibility, and metabolic predisposition are considered risk factors for this disease. Degeneration is mainly characterized by the destruction of articular cartilage, which is composed of abundant extracellular matrix (ECM) that is rich in sulfated proteoglycan and type II collagen [1]. In OA, synovitis is believed to be a reactive process as a result of cartilage destruction and the release of ECM-degradation products in the synovial fluid [1]. Loss of the ECM is caused by the secretion of degradative enzymes from chondrocytes in response to cytokines and prostaglandin E_2 _(PGE_2_) within the joint [1,2]. Matrix metalloproteinases (MMPs) are implicated in the destruction of articular cartilage in arthritis [3]. MMP-3 is believed to be a key enzyme involved in the degradation of the ECM [4]. MMPs are produced as proenzymes, which need to be activated by other enzymes such as plasmin or already activated MMPs [5]. Levels of proMMP-3 are reported to increase in joint injury and OA [6].

Fibroblast growth factor (FGF) is a family of at least 24 growth-regulatory proteins – sharing 35% to 50% amino acid sequence identity – that have potent mitogenic effects on a variety of cells of mesodermal and ectodermal origin [7,8]. FGF8 was originally isolated from the conditioned medium of an androgen-dependent mouse mammary tumor cell line (SC-3) as an androgen-induced growth factor, and was later classified as a member of the FGF family based on structural similarity [9]. Alternative splicing of the FGF8 gene potentially gives rise to eight different protein isoforms (FGF8a to FGF8h) in mice, and to four isoforms (FGF8a, FGF8b, FGF8e, and FGF8f) in humans [10,11]. FGF8b has the highest capacity among these isoforms on NIH3T3 cell transformation [12].

FGF signaling is transduced through the formation of a complex of a growth factor, a proteoglycan, and a high-affinity fibroblast growth factor receptor (FGFR), which is a transmembrane tyrosine kinase receptor [13]. Four different high-affinity receptors (FGFR1, FGFR2, FGFR3, and FGFR4) bind FGF ligands and display varying patterns of expression [14]. Extracellular domains of FGFRs consist of three immunoglobulin-like loops (loop I, loop II, and loop III). Alternative mRNA splicing of loop III of FGFR1 to FGFR3 leads to distinct functional variants (IIIb and IIIc) that have different ligand-binding specificities and affinities.

FGF8 can bind to three receptors – FGFR2IIIc, FGFR3IIIc, and FGFR4 [15] – and has an important role in embryogenesis and morphogenesis [16]. FGF8 is expressed during gastrulation and is involved in the process of limb and facial morphogenesis in mice [17] and in chicks [18]. In the complete absence of both FGF4 and FGF8, limb development fails [19]. FGF8 may also be involved in ectopic bone and cartilage formation by breast cancer cells that produce high amounts of FGF8 [20]. In addition, expression of FGF8 mRNA in the synovial sarcoma cell line has been reported [21]. The function of FGF8 in OA has not yet been characterized.

Treatment of OA patients is aimed at controlling pain, improving function, and reducing disability. The most common pharmacologic therapeutic agents used currently are analgesics, which include nonsteroidal antiinflammatory drugs and hyaluronic acid, but these drugs do not prevent the development and progression of OA.

The purpose of the present study was to examine whether FGF8 is involved in the destruction of cartilage in OA models. Initially, a rabbit meniscectomy model of OA, in which typical degenerative changes are observed in the operated knee joints [22,23], was used to detect the expression of FGF8. The activities of FGF8 were studied *in vitro *using cultures of rabbit articular chondrocytes. We also examined a neutralizing monoclonal anti-FGF8 antibody [24,25] to prevent the progression of cartilage degradation in the rat OA model, which was induced by the injection of monoiodoacetic acid (MIA) into the joint [26].

## Materials and methods

### Materials

Recombinant human FGF8 was purchased from PeproTech (Rocky Hill, NJ, USA). The anti-FGF8 neutralizing antibody, KM1334, was prepared as described previously [24,25]. KM1334 recognized FGF8b and FGF8f specifically out of the four human FGF8 isoforms, and showed little binding to other members of the FGF family. Neutralizing activity of KM1334 was shown by the blocking of FGF8b binding to its receptors. KM1334 neutralizes the activity of human FGF8, rabbit FGF8, and rat FGF8.

### Animals

All procedures were approved by the Institutional Review Board. Male New Zealand white rabbits were purchased from Kitayama Labes (Nagano, Japan). Male Sprague–Dawley rats were purchased from Charles River Japan (Kanagawa, Japan). All animals were kept in a specific pathogen-free animal facility at a temperature of 22 to 24°C, a humidity of 50% to 60%, and with a 12-hour day/night cycle.

### Partial meniscectomy model in the rabbit knee

Six male rabbits (2 to 2.5 kg) were anesthetized by intramuscular injection of ketamine (15 mg/kg) and xylazine (2 mg/kg). Each animal was subjected to section of the fibular collateral and sesamoid ligaments of the left knee and the resection of a 3 to 4 mm segment (approximately 30% to 40%) of the lateral meniscus (the meniscectomized group) according to previous reports [22,23]. In four additional animals, the ligaments were resected and the joint space of the left knee was exposed without subsequent partial meniscectomy as a sham operation. Two weeks after the surgery, the rabbits were killed by an intravenous overdose of anesthetic.

Histological evaluation was performed on sections of the synovia and articular cartilage from meniscectomized knees, from sham-operated knees (the sham group), and from nonoperated knees of the sham group (the normal group). Specimens were fixed in 10% formalin, decalcified, and embedded in paraffin. Four-micrometer sections were prepared and stained with H&E or safranin O, and were subjected to immunohistochemistry for FGF8. Histopathology of the synovial membranes of the knee joints were evaluated using the specimens stained with H&E.

Serial sections were subjected to immunohistochemistry for FGF8. These sections were treated with primary antibody (anti-FGF8 antibody KM1334 or mouse IgG_1_, 2.7 μg/ml in PBS without calcium and magnesium containing 1% BSA) overnight at 4°C and were washed with PBS without calcium and magnesium containing 1% BSA. Secondary antibody (horseradish peroxidase-labeled anti-mouse IgG rabbit polyclonal antibody (Dako Japan, Kyoto, Japan) diluted 1:200 in PBS without calcium and magnesium containing 1% BSA) was then added and incubation continued for 60 minutes at room temperature. Finally, the sections were washed, developed using diaminobenzidine, and were counterstained with hematoxylin.

The presence of proteoglycan in cartilage was assessed using the specimens stained with safranin O–fast green. The articular cartilage was graded according to the modified Mankin scale, as described elsewhere [27]. Histological evaluations were performed in a blinded manner using the following criteria: grade 0, normal; grade 1+, very slight; grade 2+, slight; grade 3+, moderate; and grade 4+, marked.

### Cell culture

Articular chondrocytes were isolated from the shoulder and knee joints of 3-week-old rabbits as previously described [28]. Cartilage was digested with 0.4% actinase E (Kaken Pharmaceuticals, Tokyo, Japan) for 1 hour at 37°C, followed by 0.025% collagenase P (Roche Diagnostics, Basel, Switzerland) for 5 hours at 37°C. The viability of the harvested cells as assessed by trypan blue exclusion was always >95%. The chondrocytes were then suspended in DMEM (Invitrogen, Carlsbad, CA, USA) with 10% FBS (Intergen, Purchase, NY, USA) and antibiotics (100 U/ml penicillin and 0.1 mg/ml streptomycin; Invitrogen). The chondrocytes were cultured in each well of 24-well tissue culture plates at a density of 1 × 10^5 ^cells/ml under 5% carbon dioxide–95% air at 37°C. Primary cultures maintained in a monolayer were used for all the experiments.

Synovial cells were collected from the knee joints of 3-week-old rabbits by the method of Hamilton and Slywka [29]. The cells were suspended in 10% FBS/DMEM with antibiotics in 80 cm^2 ^bottle flasks.

### Degradation of the extracellular matrix in chondrocyte cultures

After the chondrocytes reached confluence, the culture medium was replaced with 0.5% FBS/DMEM, followed by culturing for 24 hours. The culture medium was removed, and cells were incubated with 1 ml of 0.5% FBS/DMEM in the presence of FGF8 and/or recombinant human IL-1α (R&D Systems, Minneapolis, MN, USA) with various concentrations of anti-FGF8 antibody. A concentration of FGF8 (100 ng/ml) that caused ECM degradation was used. FGF8 at 10 ng/ml did not induce ECM degradation. A low concentration (0.01 ng/ml) of IL-1α that showed no effect by itself on ECM degradation was used. After 48 hours of incubation, the concentrations of proMMP-3 and PGE_2 _in the culture supernatant were measured using commercially available kits – proMMP-3 (Amersham Pharmacia Biotech, Piscataway, NJ, USA) and PGE_2 _(Cayman Chemical, Ann Arbor, MI, USA). The amount of sulfated glycosaminoglycan (S-GAG) in the ECM remaining on the plate was measured using 1,9-dimethylmethylene blue (Sigma-Aldrich, St Louis, MO, USA) as previously described [30].

### Growth of synovial cells

Rabbit synovial cells were collected by trypsin treatment, suspended in 10% FBS/RPMI 1640 medium, and cultured in each well of 96-well tissue culture plates at a density of 10,000 cells/well under 5% carbon dioxide–95% air at 37°C. After 24 hours the culture medium of each well was removed, and then 0.2% FBS/RPMI 1640 medium (200 μl) with or without FGF8 and/or various concentrations of anti-FGF8 antibody were added to the cells. After 48 hours, 9.25 kBq methyl [^3^H]thymidine (Amersham Pharmacia Biotech) was added to each well. The radioactivity of [^3^H]thymidine incorporated into the cells was measured 24 hours after the incubation using a liquid scintillation counter (1205 Beta Plate; Perkin Elmer Japan, Kanagawa, Japan).

### Intraarticular injection of FGF8 in rats

Arthritis-like symptoms were induced in 7-week-old rats using FGF8 as follows. FGF8 (50 μg/site, 50 μl of 1 mg/ml solution in sterile saline) was injected into the right knee joint of each rat (the FGF8 group). The anti-FGF8 antibody KM1334 was dissolved in sterile saline at a concentration of 4 mg/ml. KM1334 (20 mg/kg) was administered by intraperitoneal injection 1 hour before the injection of FGF8 (the anti-FGF8 antibody group). For the vehicle group, sterile saline was administered intraperitoneally instead of the antibody solution. For the saline group, 50 μl sterile saline was injected into the knee joint and sterile saline was administered intraperitoneally instead of the antibody solution. Each group consisted of five rats.

After 3 days, the inside of the knee articular capsule was washed with 30 μl saline containing 0.38% sodium citrate, and this washing liquid (synovial lavage fluid) was collected according to the method of Yamada and colleagues [31]; this procedure was repeated 10 times. The amount of S-GAG in an aliquot was measured by the 1,9-dimethylmethylene blue method. The patella of the knee joint was taken out and the cartilaginous portion was digested with papain, and the weight of the residual bone was measured according to a previous report [32]. Briefly, papain was dissolved in 0.1 M sodium acetate buffer (pH 5.8) containing 50 mM ethylenediamine tetraacetic acid and then added to 5 mM L-cysteine hydrochloride monohydrate (final concentration of papain at 20 mg/ml). The patella was incubated with 1 ml papain solution overnight at 60°C. The residual bone was weighed.

### Monoiodoacetic acid-induced arthritis in rats

The MIA-induced rat arthritis model was performed according to previous reports [26]. MIA (Sigma-Aldrich) was dissolved at 10 mg/ml in sterile saline, and 25 μl solution was injected into the right knee joint of 7-week-old rats. Each group consisted of 10 animals. The anti-FGF8 antibody KM1334 was dissolved in sterile saline to a final concentration of 4 mg/ml, and was administered by intraperitoneal injection (20 mg/kg) 1 hour before the injection of MIA. For the vehicle group, sterile saline was administered intraperitoneally instead of the antibody solution. In a saline group of animals, saline was injected in the knee joint instead of the administration of MIA. After 3 days, synovial lavage was performed to the knee articular capsule as described above. The amount of S-GAG in the synovial lavage fluid was measured by the 1,9-dimethylmethylene blue method.

### Statistical analysis

Data are presented as the mean ± standard error. The Aspin–Welch test or Student's *t *test following the *F *test were used for analysis of difference between two groups. Multiple comparisons between control and treatment groups were assessed by one-way analysis of variance, followed by the Dunnett test. *P *< 0.05 was considered statistically significant. All statistical calculations were performed with the Statistical Analysis System (SAS Institute, Cary, NC, USA).

## Results

### Expression of FGF8 in the partial meniscectomized experimental osteoarthritis model

**Figure 1:**
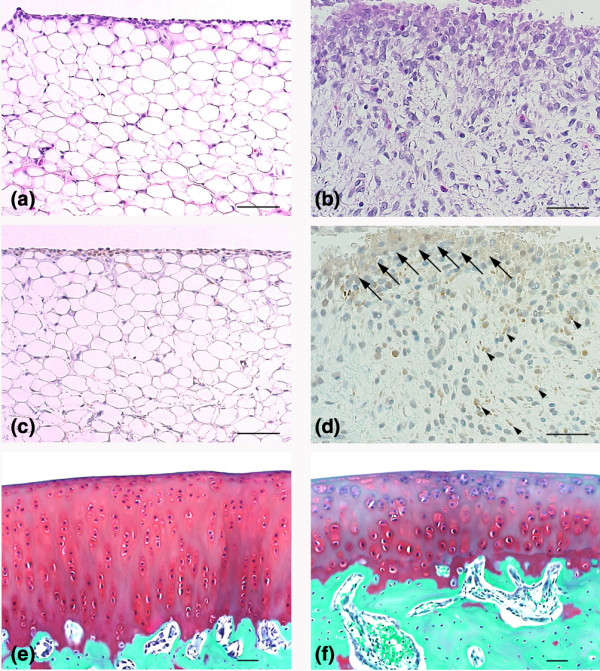
Microphotographs of representative knee joints of meniscectomized rabbits. Partial meniscectomy was performed on the rabbit knee to induce osteoarthritis-like morphology. Representative histologic sections of synovial membrane in **(a) **an untreated knee joint (normal) or **(b) **a meniscectomized knee joint were stained with H&E. Hyperplasia of synovial cells, fibrosis, and inflammatory cell infiltration was observed in the meniscectomized knee. Synovial membrane in **(c) **a normal knee joint or (b) a meniscectomized knee joint were immunostained with the anti-FGF8 antibody KM1334. Sections were reacted with horseradish peroxidase-labeled anti-mouse IgG, developed using diaminobenzidine (brown) and counter stained with hematoxylin (blue). Very weak staining of FGF8 was observed in a few synovial cells, but not in other tissues in normal knee joints. **(d) **Many of the proliferated synovial cells (arrows) in meniscectomized knee joints showed positive reaction to FGF8. Positive staining of FGF8 was also observed in fibroblasts (arrowheads). **(e) **Normal and **(f) **meniscectomized articular cartilage was stained with safranin O. Reduction of safranin-O staining (red) and clusters of chondrocyte were observed in meniscectomized articular cartilage. Scale bar = 20 μm.

Arthritis was induced by partial meniscectomy in the rabbit knee. Two weeks after the surgery, an accumulation of synovial fluid was observed in the joint space of the meniscectomized group. A representative histologic change of the meniscectomized joint is shown in Figure 1. Hyperplastic proliferation of synovial cells, fibrosis, and infiltration of inflammatory cells were observed in the joint space of meniscectomized knees (Figure 1b and Table 1). In the normal group, no histological changes were observed (Figure 1a and Table 1). In the meniscectomized group, synovial cells with positive staining for FGF8 (Figure 1d, arrows) were increased as compared with the normal group (Figure 1c and Table 1).

**Table 1:** Histological findings of knee joints in the meniscectomized rabbit osteoarthritis model

Histological findings of synovium^a^	Meniscectomy group (*n* = 6)					Sham group (*n *= 4)					Normal group (*n* = 4)				
	
	0	1+	2+	3+	4+	0	1+	2+	3+	4+	0	1+	2+	3+	4+
H&E staining															
Hyperplasia of synovial cell	0	0	5	1	0	1	0	3	0	0	4	0	0	0	0
Fibrosis in connective tissue	0	0	1	5	0	0	0	1	3	0	4	0	0	0	0
Inflammatory cell infiltration in connective tissue	4	0	1	1	0	4	0	0	0	0	4	0	0	0	0
KM1334 immunohistochemistry^b^															
Positive reactions in synovial cells	0	6	0	0	0	1	3	0	0	0	3^c^	1	0	0	0
Positive reactions in fibroblastic cells	0	5	1	0	0	0	4	0	0	0	4	0	0	0	0
Positive reactions in cartilage	6^c^	0	0	0	0	4	0	0	0	0	4^c^	0	0	0	0
Cartilage degeneration score^c ^(mean ± standard error)	6.0 ± 0.8					0.5 ± 0.5					0.0 ± 0.0				

^a^Histological findings criteria: 0, non remarkable; 1+, very slight; 2+, slight, 3+, moderate; 4+, marked. ^b^KM1334; an anti-FGF8 antibody. ^c^Safranin O/fast green staining sample examined using the modified Mankin scale.

FGF8 was expressed in the fibroblasts after meniscectomy (Figure 1d, arrowheads). In the normal group, very weak expression of FGF8 was observed in synovial cells but not in fibroblasts (Figure 1c). No positive reaction for FGF8 was observed in the articular cartilage from both the normal group and the meniscectomized group (Table 1). In the sham group, synovial cells showed a weak positive reaction for FGF8 in three out of four animals, and fibroblasts also showed a weak positive reaction in all animals (Table 1). These findings suggest that the expression of FGF8 was induced in synovia and fibroblasts by mechanical injury.

Representative sections of cartilage in the normal group (Figure 1e) and in the meniscectomized group (Figure 1f) were stained by safranin O. Reduction of cartilage and the presence of clusters of chondrocytes occurred in the meniscectomized group (Figure 1f). The severity of histological changes of the articular cartilage was evaluated using a modified Mankin scale (Table 1). The mean scores of the Mankin scale for the meniscectomized group, for the sham group, and for the normal group were 6.0 ± 0.8, 0.5 ± 0.5, and 0.0 ± 0.0, respectively.

### Degradation of the extracellular matrix of chondrocytes by FGF8

**Figure 2:**
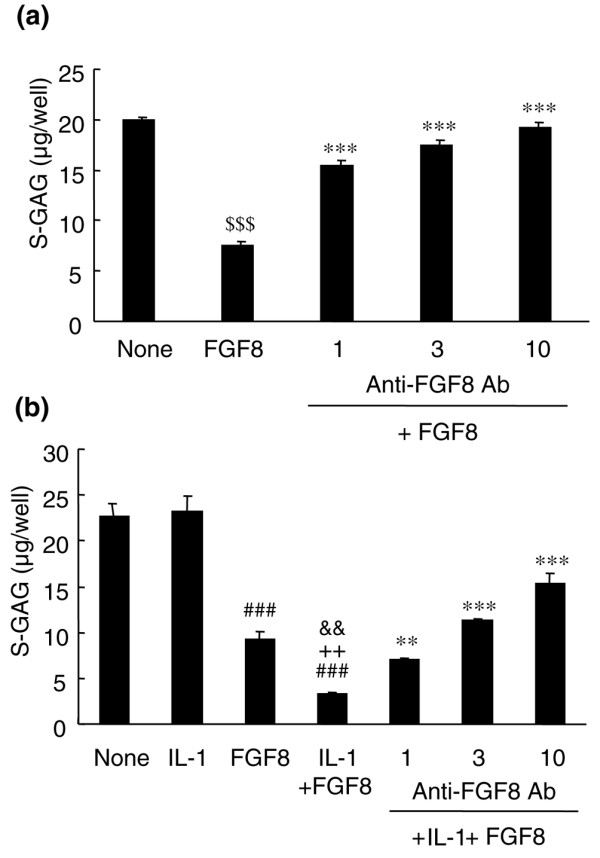
FGF8 induced the decrease in the sulfated glycosaminoglycan content in the cellular matrix. Chondrocytes were treated without (none) or with 100 ng/ml FGF8 and/or 0.01 ng/ml IL-1α and various concentrations of anti-FGF8 antibody (Ab) for 48 hours. Sulfated glycosaminoglycan (S-GAG) content of the residual cellular matrix was measured using the 1,9-dimethylmethylene blue method. Each column represents the mean ± standard error. **(a) **FGF8 induced S-GAG degradation, which was concentration-dependently inhibited by anti-FGF8 antibody (1, 3 and 10 μg/ml). Representative data of three independent experiments. ^$$$^*P *< 0.001 compared with the no treatment group (Student's *t *test), ***P < 0.001 compared with FGF8 alone (Dunnett test). **(b) **Chondrocytes were treated with IL-1α (IL-1), FGF8, or IL-1α with FGF8 (IL-1 + FGF8). IL-1α enhanced FGF8-induced S-GAG degradation (IL-1 + FGF8). Anti-FGF8 antibody (1, 3 and 10 μg/ml) concentration-dependently inhibited that degradation by FGF8 with IL-1α. Data from a single experiment are shown, but similar data were obtained in two additional experiments. ^###^*P *< 0.001 compared with the no treatment group (Student's *t *test), ^++^*P *< 0.01 compared with IL-1α alone (Aspin–Welch test), ^&&^*P *< 0.01 compared with FGF8 alone (Student's *t *test), **P < 0.01 and ***P < 0.001 compared with the IL-1α + FGF8-treated group (Dunnett test).

To elucidate the role of FGF8 in meniscectomy-induced cartilage destruction, we investigated the activities of FGF8 on the cultured chondrocytes. FGF8 dose-dependently induced ECM degradation. The residual amounts of S-GAG in cultured rabbit chondrocytes were 22.3, 21.0, 20.1, and 9.28 μg/well at 0, 1, 10, and 100 ng/ml FGF8, respectively. The degradation of the ECM by 100 ng/ml FGF8 was inhibited dose-dependently by anti-FGF8 antibody at 1 to 10 μg/ml (Figure 2a).

IL-1 is one of the important factors that promote cartilage degradation. In the absence of FGF8, S-GAG in the ECM was not decreased by the addition of a low concentration of IL-1α (0.01 ng/ml). Upon incubation with IL-1α (0.01 ng/ml) and FGF8 (100 ng/ml) for 48 hours, there was a significant decrease in residual S-GAG compared with levels after FGF8 stimulation or IL-1α stimulation alone (Figure 2b). The enhancement of IL-1α-induced S-GAG release by FGF8 was concentration-dependently suppressed by the addition of anti-FGF8 antibody at 1 to 10 μg/ml (Figure 2b).

### Stimulation of proMMP-3 and prostaglandin E_2 _production by FGF8 in rabbit chondrocytes

**Figure 3:**
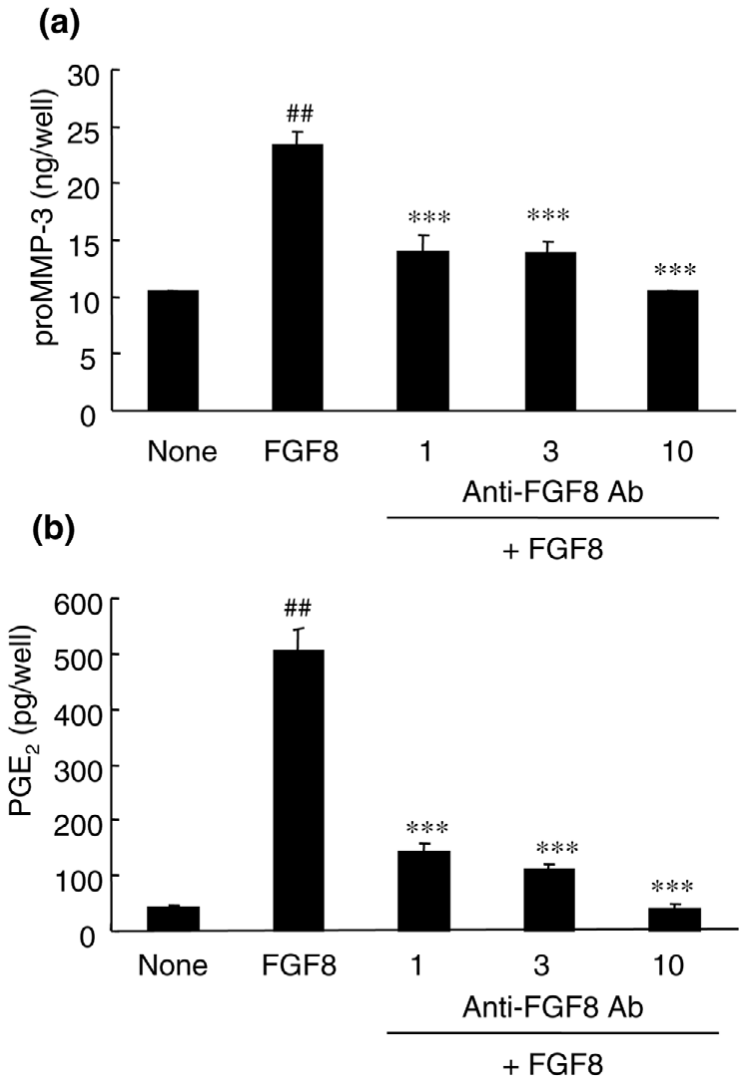
FGF8 enhanced the release of promatrix metalloproteinase-3 and prostaglandin E_2 _from rabbit articular chondrocyte cultures. Chondrocytes were treated without (none) or with 100 ng/ml FGF8 and various concentrations of anti-FGF8 antibody (Ab) (1, 3 and 10 μg/ml) for 48 hours. Concentrations of **(a) **promatrix metalloproteinase-3 (proMMP-3) and **(b) **prostaglandin E_2 _(PGE_2_) in the culture medium were determined by ELISA. Each column represents the mean ± standard error. Representative data of two or three independent experiments. ^##^*P *< 0.01 compared with the no treatment group (Aspin–Welch test), ***P < 0.001 compared with FGF8 alone (Dunnett test).

The effect of FGF8 on the release of proMMP-3 and PGE_2 _in the culture medium was investigated. In the presence of FGF8 (100 ng/ml), production of proMMP-3 by rabbit chondrocytes was significantly induced (*P *= 0.0079; Figure 3a). This indicated that FGF8 may promote degradation of the ECM by production of proteases including MMP-3. When anti-FGF8 antibody was added to the culture at 1 to 10 μg/ml, there was a significant inhibition of proMMP-3 production.

PGE_2 _is a mediator of inflammation and pain. FGF8 significantly increased the production of PGE_2 _from the chondrocytes (*P *= 0.0063; Figure 3b). The production of PGE_2_, which was induced by FGF8, decreased dose-dependently in the presence of anti-FGF8 antibody at 1 to 10 μg/ml (Figure 3b).

### Promotion of growth of rabbit synovial cells by FGF8

**Figure 4:**
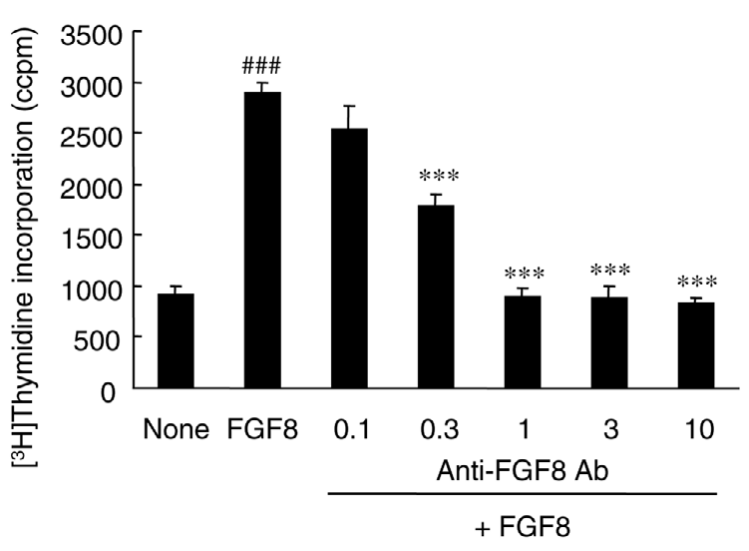
FGF8 induced the growth of rabbit synovial fibroblast-like cells. Rabbit synovial fibroblast-like cells were treated without (none) or with 100 ng/ml FGF8 and various concentrations of anti-FGF8 antibody (Ab) (0.1, 0.3, 1, 3 and 10 μg/ml) for 72 hours. [^3^H]Thymidine incorporation during the last 24-hour pulse of cultures was determined. Each column represents the mean ± standard error. Data from a single experiment are shown, but similar data were obtained in three additional experiments. ^###^*P *< 0.001 compared with the no treatment group (Student's *t *test), ***P < 0.001 compared with FGF8 alone (Dunnett test).

The effects of FGF8 on synovial cells were assessed using primary cultures of rabbit synovial cells. FGF8 at 100 ng/ml significantly promoted the growth of rabbit synovial cells as measured by the incorporation of [^3^H]thymidine (Figure 4). FGF8 at 100 ng/ml significantly promoted incorporation of [^3^H]thymidine more than threefold compared with nonstimulated cells (*P *< 0.0001). The addition of anti-FGF8 antibody at 0.3 to 10 μg/ml significantly inhibited FGF8-induced incorporation of [^3^H]thymidine.

### Articular destruction by intraarticular injection of FGF8 into the rat knee joint

**Figure 5:**
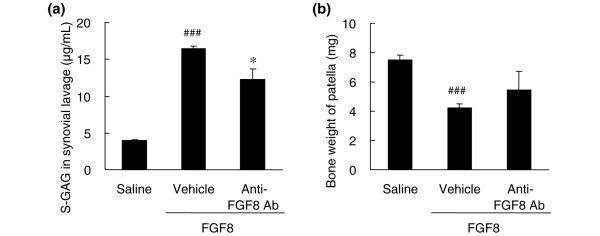
Intraarticular injection of FGF8 induced the sulfated glycosaminoglycan release in knee joints and destruction of patella. Fifty micrograms of FGF8 was injected into the knee joint of rats (vehicle). KM1334 (the anti-FGF8 antibody (Ab)) was intraperitoneally administered before the injection of FGF8. For the saline injection group (saline), 50 μl sterile saline was injected into the knee joint. Synovial lavage was performed with saline 3 days after the FGF8 injection. **(a) **Sulfated glycosaminoglycan (S-GAG) content in the synovial lavage fluid was measured by the 1,9-dimethylmethylene blue method. **(b) **The cartilaginous portion of the patella was digested with papain to measure the weight of the residual bone. Each column represents the mean ± standard error (*n* = 5). ^###^*P *< 0.001 compared with the saline group (Student's *t *test), *P < 0.05 compared with the vehicle group (Aspin–Welch test).

The activity of FGF8 on the knee joint was tested by the articular injection of FGF8 to rats. FGF8 dose-dependently increased the release of S-GAG in joint fluid. The concentrations of S-GAG were 3.96, 6.35, 12.2, and 16.5 μg/ml by 0, 0.5, 5, and 50 μg/site FGF8, respectively. An injection of 50 μg/site FGF8 increased the concentration of S-GAG in joint fluid (Figure 5a). The amount of S-GAG in the FGF8 injection group was 4.2 times higher than that of the saline injection group (*P *< 0.0001). Anti-FGF8 antibody significantly inhibited the degradation of GAG in the FGF8-treated joints by 33% (*P *= 0.036). These data indicate that the injection of FGF8 causes degradation of the ECM of the articular cartilage and release of S-GAG into the synovial fluid. In addition, the injection of FGF8 decreased the bone weight of the patella to 43% of the saline injection group (*P *< 0.0001; Figure 5b). Anti-FGF8 antibody attenuated bone loss in the FGF8-treated joints by 34%, although this was not statistically significant.

### Evaluation of anti-FGF8 antibody in a monoiodoacetic acid-induced rat arthritis model

**Figure 6:**
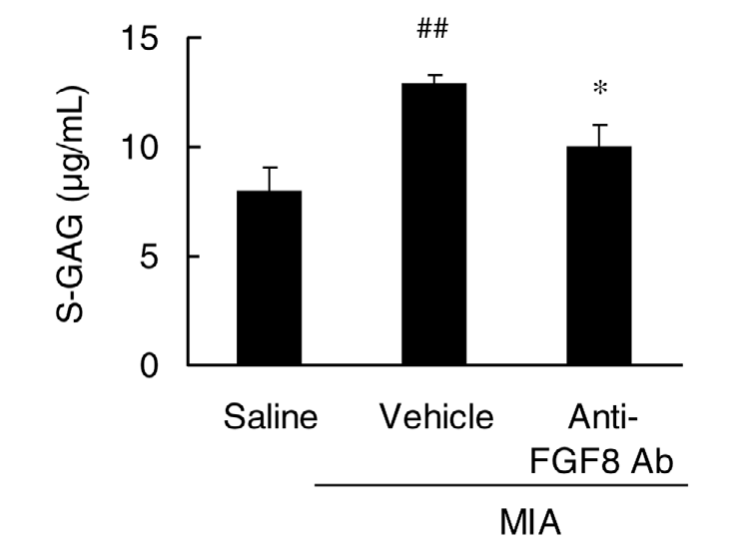
Effect of FGF8 on monoiodoacetic acid-induced sulfated glycosaminoglycan release in the knee joint of rats. Monoiodoacetic acid (MIA) was injected into the right knee joint of rats (vehicle). KM1334 (the anti-FGF8 antibody (Ab)) was intraperitoneally administered before the injection of MIA. As a control, sterile saline was injected into the knee instead of MIA (saline). The synovial lavage was performed with saline 3 days after the intraarticular injection of MIA into knee joints. The content of sulfated glycosaminoglycan (S-GAG) in an aliquot was measured by the 1,9-dimethylmethylene blue method. Each column represents the mean ± standard error (*n* = 10). ^##^*P *< 0.01 compared with the sham group (Aspin–Welch test), *P < 0.05 compared with the vehicle group (Aspin–Welch test).

Injection of MIA, an inhibitor of glycolysis, into the femorotibial joint of rats promotes loss of articular cartilage similar to that noted in human OA. The injection of MIA increased the concentration of S-GAG in the joint space of rats (Figure 6). The amount of S-GAG release in the MIA group was 1.6 times higher than that in the saline injection group (*P *= 0.0014; Figure 6). These data show that MIA promotes the degradation of the ECM of the articular cartilage and the release of S-GAG into synovial fluid. Intraperitoneal administration of anti-FGF8 antibody significantly inhibited the increase in S-GAG in the joint by 42% (*P *= 0.0188; Figure 6).

## Discussion

The present study provides new and interesting findings about the role of FGF8 in joint inflammation. We analyzed the rabbit knee in a partial meniscectomized model to clarify the expression of FGF8 – which was very slightly expressed or not expressed in normal joints. Two weeks after meniscectomy, the cartilage from the lateral femoral condyle and from the lateral tibial plateau of meniscectomized animals showed degenerative changes that were similar to those observed in human OA. In the meniscectomized group, FGF8 was expressed on proliferated synovial cells and fibroblasts, but expression of FGF8 was absent or minimal in normal joints. Weak expression of FGF8 was also exhibited in the joints in which the ligaments were resected and the articular spaces were exposed (the sham group). These data indicate that expression of FGF8 is induced by chronic joint injury.

Degradation of the ECM was promoted in the presence of FGF8. The function of FGF8 on cartilage destruction was examined using a primary culture of rabbit chondrocytes. Rabbit articular chondrocytes are useful to detect biological responses. In the present study, we used primary culture of chondrocytes from 3-week-old rabbits according to the previous report [33]. IL-1-induced matrix degradation according to the induction of metalloproteases was reported in cultured chondrocytes from 300 to 500 g young rabbit [33]. Articular chondrocytes from 4-week-old rabbits underwent more doubling *in vitro *compared with those from 3.5-year-old adult rabbits, but showed no difference in shape at primary culture [34]. FGF8 induced the production of proMMP-3 by cultured chondrocytes, and anti-FGF8 antibody attenuated the release of these factors. MMP-3 plays an important role in cartilage degradation [3,4] and is a major factor in the catabolism of cartilage macromolecules. MMP-3 resolves proteoglycan in the ECM and also activates the other MMPs that participate in the degradation of the matrix in joints. MMP-3 is increased both in cartilage and in synovial membranes from OA patients [35]. In our study, FGF8 induced the production of proMMP-3 and significantly decreased the residual amount of S-GAG in the ECM in cultured chondrocytes. These data indicate that FGF8 can promote the destruction of articular cartilage by the induction of factors such as MMP-3 and PGE_2 _in joints.

FGF8 and IL-1 synergistically accelerated the degradation of the ECM. The participation of synovial inflammation in the progression of cartilage changes at the clinical stage of OA is becoming increasingly obvious [1]. A number of clinical studies demonstrate a clear association between inflammation and disease progression [36]. A large number of inflammatory factors, such as IL-1 and TNFα, are synthesized within inflamed synovium and play an important role in articular destruction in OA [1]. In cultured chondrocytes, degradation of the ECM induced by FGF8 was further enhanced by a small amount of IL-1. Anti-FGF8 antibody blocked this FGF8 with IL-1-induced degradation of the ECM. IL-1 causes both matrix degradation and downregulation of proteoglycan synthesis [37], but further studies are required to clarify whether FGF8 also downregulates ECM synthesis.

FGF8 induced the production of PGE_2 _from cultured chondrocytes. PGE_2 _is the major prostaglandin in the synovial fluid of OA patients, and is produced by IL-1-stimulated chondrocytes and synoviocytes from OA patients [37]. Cartilage specimens from OA patients spontaneously release more PGE_2 _than does normal cartilage [38]. Nonsteroidal antiinflammatory drugs, including cyclooxygenase-2 inhibitors, are commonly used to control pain and inflammation in OA [1]. These drugs inhibit the production of PGE_2 _and consequently relieve pain caused by PGE_2_. In the present study, a low concentration of anti-FGF8 antibody markedly inhibited the production of PGE_2 _by cultured chondrocytes. Anti-FGF8 antibody might be expected to provide an analgesic effect because it inhibits PGE_2 _production by chondrocytes.

FGF8 induced joint destruction *in vivo*. Injection of FGF8 into the rat knee joints promoted the release of S-GAG from cartilage into the synovial fluid. Following injection of FGF8, the weight of the patella was reduced. Further study is required to determine whether FGF8 induces morphological change as shown in OA. Many of the etiologic factors responsible for OA are related to the breakdown of extracellular macromolecules. FGFs are one of the candidates that cause progression of OA [39]. FGF2 has various physiological effects on bone and cartilage metabolism [40]. FGF2, which is expressed ubiquitously in mesodermal and neuroectodermal cells, has various physiological functions. In the present study, we have demonstrated that FGF8 is selectively expressed in injured joints. Cartilage degradation is induced by exogenous FGF8. These results indicate that FGF8 is one of the selective mediators of arthritis. FGF8 is involved in the process of limb and facial morphogenesis [17]. Further studies are required of whether FGF8 has a physiological function in maturing of joints and diseases in children such as juvenile rheumatoid arthritis.

FGF8 is expressed in synovial cells and concentration-dependently enhances growth of cultured synovial cells. It is possible that FGF8 promotes growth of synovial cells via autocrine signaling. FGF8 also induced degradation of the ECM in cultured chondrocytes. These studies suggest that the injury of synovia induces the expression of FGF8 in the joint, and this may promote degradation of cartilage via a paracrine system. The bone weight of the patella was decreased following an injection of FGF8 into the rat joint. Synovial hyperplasia is known to initiate bone and cartilage erosions [1]. Bone degradation following intraarticular injection of FGF8 may therefore be due to the growth of synovial cells and also production of various factors from synoviocytes, fibroblasts, or other cells. FGF8 can induce cartilage and bone degradation to cause the arthritis-like syndromes and possibly aggravate the pathology of OA. Other factors such as mechanical stress, cytokines, and inflamed cells are also important for cartilage degeneration. Further studies are required to elucidate the contribution of FGF8 on bone absorption and joint destruction.

Anti-FGF8 antibody not only reduced cartilage degradation induced by the injection of FGF8 in the joints, but also decreased cartilage degradation in the MIA-induced rat arthritis model. These data indicate that systemic application of anti-FGF8 antibody protects the FGF8-dependent cartilage degradation. The use of MIA to chemically induce degenerative arthritis was first described by Kalbhen and Blum [41], and subsequently by other investigators [26]. The injection of MIA into the knees of rats provides a model where lesions resemble some aspects of human OA. The amounts of MMP were significantly elevated after the injection of MIA. This model has been used for the development of chondroprotective drugs [26]. Anti-FGF8 antibody inhibited release of S-GAG in the MIA-induced arthritis model. Anti-FGF8 antibody suppressed the production of proMMP-3 and PGE_2 _from cultured chondrocytes. These findings provide evidence for the potential use of anti-FGF8 antibody in the treatment of articular tissue degradation and pain in OA. An anti-FGF8 antibody is expected to attenuate the symptoms of rheumatoid arthritis. We are studying the effects of anti-FGF8 antibody on collagen-induced arthritis and on adjuvant-induced arthritis.

## Conclusion

We have demonstrated that the expression of FGF8 on synovia is increased in an experimental model of OA in rabbits. FGF8 induced production of MMP-3 and PGE_2_, and caused degradation of the ECM *in vitro*. Degradation of S-GAG was detected by intraarticular injection of FGF8 in rats. Anti-FGF8 antibody attenuates the destruction of cartilage in the MIA-induced arthritis model. These data indicate that FGF8 has a possible pathophysiological role in the degradation of cartilage in OA models.

## Abbreviations

BSA = bovine serum albumin; DMEM = Dulbecco's modified Eagle's medium; ECM = extracellular matrix; FBS = fetal bovine serum; FGF = fibroblast growth factor; FGFR = fibroblast growth factor receptor; H&E = hematoxylin and eosin; IL = interleukin; MIA = monoiodoacetic acid; MMP = matrix metalloproteinase; OA = osteoarthritis; PBS = phosphate-buffered saline; PGE_2 _= prostaglandin E_2_; S-GAG = sulfated glycosaminoglycan.

## Competing interests

MU, TT, TS and IM are employees of Kyowa Hakko Kogyo. MK was an employee of Kyowa Hakko Kogyo. MU, TT, TS, AT and IM applied for a patent of an anti-FGF8 antibody for treatment of OA (WO2003/057251). IM has stock in Kyowa Hakko Kogyo.

## Authors' contributions

IM is the principal researcher and developed the original idea for the study. The experimental study was designed and carried out by MU, TT, and TS. Pathological analysis was performed by MK. AT provided information on FGF8 and reviewed the studies. All authors read and corrected draft versions of the manuscript and approved the final version.

## References

[B1] Pelletier JP, Martel-Pelletier J, Abramson SB (2001). Osteoarthritis, an inflammatory disease: potential implication for the selection of new therapeutic targets. Arthritis Rheum.

[B2] Hardy MM, Seibert K, Manning PT, Currie MG, Woerner BM, Edwards D, Koki A, Tripp CS (2002). Cyclooxygenase 2-dependent prostaglandin E_2 _modulates cartilage proteoglycan degradation in human osteoarthritis explants. Arthritis Rheum.

[B3] Lohmander LS, Hoerrner LA, Lark MW (1993). Metalloproteinases, tissue inhibitor, and proteoglycan fragments in knee synovial fluid in human osteoarthritis. Arthritis Rheum.

[B4] Okada Y, Shinmei M, Tanaka O, Naka K, Kimura A, Nakanishi I, Bayliss MT, Iwata K, Nagase H (1992). Localization of matrix metalloproteinase 3 (storomelysin) in osteoarthritic cartilage and synovium. Lab Invest.

[B5] Nagase H (1997). Activation mechanisms of matrix metalloproteinases. Biol Chem.

[B6] Tchetverikov I, Lohmander LS, Verzijl N, Huizinga TW, TeKoppele JM, Hanemaaijer R, DeGroot J (2005). MMP protein and activity levels in synovial fluid from patients with joint injury, inflammatory arthritis, and osteoarthritis. Ann Rheum Dis.

[B7] Szebenyi G, Fallon JF (1999). Fibroblast growth factors as multifunctional signaling factors. Int Rev Cytol.

[B8] Draper BW, Stock DW, Kimmel CB (2003). Zebrafish fgf24 functions with fgf8 to promote posterior mesodermal development. Development.

[B9] Tanaka A, Miyamoto K, Minamino N, Takeda M, Sato B, Matsuo H, Matsumoto K (1992). Cloning and characterization of an androgen-induced growth factor essential for the androgen-dependent growth of mouse mammary carcinoma cells. Proc Natl Acad Sci USA.

[B10] MacArthur CA, Lawshe A, Xu J, Santos-Ocampo S, Heikinheimo M, Chellaiah AT, Ornitz DM (1995). FGF-8 isoforms activate receptor splice forms that are expressed in mesenchymal regions of mouse development. Development.

[B11] Gemel J, Gorry M, Ehrlich GD, MacArthur CA (1996). Structure and sequence of human FGF8. Genomics.

[B12] MacArthur CA, Lawshe A, Shankar DB, Heikinheimo M, Shackleford GM (1995). FGF-8 isoforms differ in NIH3T3 cell transforming potential. Cell Growth Differ.

[B13] McKeehan WL, Wang F, Kan M (1998). The heparan sulfate-fibroblast growth factor family: diversity of structure and function. Prog Nucleic Acid Res Mol Biol.

[B14] Ornitz DM, Xu J, Colvin JS, McEwen DG, MacArthur CA, Coulier F, Gao G, Goldfarb M (1996). Receptor specificity of the fibroblast growth factor family. J Biol Chem.

[B15] Blunt AG, Lawshe A, Cunningham ML, Seto ML, Ornitz DM, MacArthur CA (1997). Overlapping expression and redundant activation of mesenchymal fibroblast growth factor (FGF) receptors by alternatively spliced FGF-8 ligands. J Biol Chem.

[B16] Ohuchi H, Yoshioka H, Tanaka A, Kawakami Y, Nohno T, Noji S (1994). Involvement of androgen-induced growth factor (FGF-8) gene in mouse embryogenesis and morphogenesis. Biochem Biophys Res Commun.

[B17] Mahmood R, Bresnick J, Hornbruch A, Mahony C, Morton N, Colquhoun K, Martin P, Lumsden A, Dickson C, Mason I (1995). A role for FGF-8 in the initiation and maintenance of vertebrate limb bud outgrowth. Curr Biol.

[B18] Crossley PH, Minowada G, MacArthur CA, Martin GR (1996). Roles for FGF8 in the induction, initiation, and maintenance of chick limb development. Cell.

[B19] Sun X, Mariani FV, Martin GR (2002). Functions of FGF signalling from the apical ectodermal ridge in limb development. Nature.

[B20] Valta MP, Hentunen T, Qu Q, Valve EM, Harjula A, Seppanen JA, Vaananen HK, Harkonen PL (2006). Regulation of osteoblast differentiation: a novel function for fibroblast growth factor 8. Endocrinology.

[B21] Ishibe T, Nakayama T, Okamoto T, Aoyama T, Nishijo K, Shibata KR, Shima Y, Nagayama S, Katagiri T, Nakamura Y, Nakamura T, Toguchida J (2005). Disruption of fibroblast growth factor signal pathway inhibits the growth of synovial sarcomas: potential application of signal inhibitors to molecular target therapy. Clin Cancer Res.

[B22] Kikuchi T, Yamada H, Shinmei M (1996). Effect of high molecular weight hyaluronan on cartilage degeneration in a rabbit model of osteoarthritis. Osteoarthr Cartil.

[B23] Colombo C, Butler M, O' Byrne E, Hickman L, Swartzendruber D, Selwyn M, Steinetz B (1983). A new model of osteoarthritis in rabbits. I. Development of knee joint pathology following lateral meniscectomy and section of the fibular collateral and sesamoid ligaments. Arthritis Rheum.

[B24] Tanaka A, Furuya A, Yamasaki M, Hanai N, Kuriki K, Kamiakito T, Kobayashi Y, Yoshida H, Koike M, Fukayama M (1998). High frequency of fibroblast growth factor (FGF) 8 expression in clinical prostate cancers and breast tissues, immunohistochemically demonstrated by a newly established neutralizing antibody against FGF 8. Cancer Res.

[B25] Shimada N, Ishii T, Imada T, Takaba K, Sasaki Y, Maruyama-Takahashi K, Maekawa-Tokuda Y, Kusaka H, Akinaga S, Tanaka A, Shitara K (2005). A neutralizing anti-fibroblast growth factor 8 monoclonal antibody shows potent antitumor activity against androgen-dependent mouse mammary tumors *in vivo*. Clin Cancer Res.

[B26] Janusz MJ, Hookfin EB, Heitmeyer SA, Woessner JF, Freemont AJ, Hoyland JA, Brown KK, Hsieh LC, Almstead NG, De B, Natchus MG, Pikul S, Taiwo YO (2001). Moderation of iodoacetate-induced experimental osteoarthritis in rats by matrix metalloproteinase inhibitors. Osteoarthr Cartil.

[B27] Kuroki H, Nakagawa Y, Mori K, Ohba M, Suzuki T, Mizuno Y, Ando K, Takenaka M, Ikeuchi K, Nakamura T (2004). Acoustic stiffness and change in plug cartilage over time after autologous osteochondral grafting: correlation between ultrasound signal intensity and histological score in a rabbit model. Arthritis Res Ther.

[B28] Tamura T, Kosaka N, Ishiwa J, Sato T, Nagase H, Ito A (2001). Rhein, an active metabolite of diacerein, down-regulates the production of pro-matrix metalloproteinases-1, -3, -9 and -13 and up-regulates the production of tissue inhibitor of metalloproteinase-1 in cultured rabbit articular chondrocytes. Osteoarthr Cartil.

[B29] Hamilton JA, Slywka J (1981). Stimulation of human synovial fibroblast plasminogen activator production by mononuclear cell supernatants. J Immunol.

[B30] Chandrasekhar S, Esterman MA, Hoffman NA (1987). Microdetermination of proteoglycan and glycosaminoglycans in the presence of guanidine hydrochloride. Anal Biochem.

[B31] Yamada A, Uegaki A, Makamura T, Ogawa K (2000). ONO – an orally active matrix metalloproteinase inhibitor, prevents lipopolysaccharide-induced proteoglycan release from the joint cartilage in guinea pigs. Inflamm Res.

[B32] Schrier DJ, Flory CM, Finkel M, Kuchera SL, Lesch ME, Jacobson PB (1996). The effects of the phospholipase A_2 _inhibitor, manoalide, on cartilage degradation, stromelysin expression, and synovial fluid cell count induced by intraarticular injection of human recombinant interleukin-1 in the rabbit. Arthritis Rheum.

[B33] Tamura T, Nakanishi T, Kimura Y, Hattori T, Sasaki K, Norimatsu H, Takahashi K, Takigawa M (1996). Nitric oxide mediates interleukin-1-induced matrix degradation and basic fibroblast growth factor release in cultured rabbit articular chondrocytes: a possible mechanism of pathological neovascularization in arthritis. Endocrinology.

[B34] Evans CH, Georgescu HI (1983). Observations on the senescence of cells derived from articular cartilage. Mech Ageing Dev.

[B35] Stove J, Gerlach C, Huch K, Gunther KP, Brenner R, Puhl W, Scharf HP (2001). Gene expression of stromelysin and aggrecan in osteoarthritic cartilage. Pathobiology.

[B36] Ayral X, Pickering EH, Woodworth TG, Mackillop N, Dougados M (2005). Synovitis a potential predictive factor of structural progression of medial tibiofemoral knee osteoarthritis – results of a 1 year longitudinal arthroscopic study in 422 patients. Osteoarthritis Cartilage.

[B37] Arner EC, Pratta MA (1989). Independent effects of interleukin-1 on proteoglycan breakdown, proteoglycan synthesis, and prostaglandin E_2 _release from cartilage in organ culture. Arthritis Rheum.

[B38] Amin AR, Attur M, Patel RN, Thakker GD, Marshall PJ, Rediske J, Stuchin AS, Patel IR, Abramson SB (1997). Superinduction of cyclooxygenase-2 activity in human osteoarthritis-affected cartilage: influence of nitric oxide. J Clin Invest.

[B39] Daouti S, Latario B, Nagulapalli S, Buxton F, Uziel-Fusi S, Chirn GW, Bodian D, Song C, Labow M, Lotz M, Quintavalla J, Kumar C (2005). Development of comprehensive functional genomic screens to identify novel mediators of osteoarthritis. Osteoarthr Cartil.

[B40] Chuma H, Mizuta H, Kudo S, Takagi K, Hiraki Y (2004). One day exposure to FGF-2 was sufficient for the regenerative repair of full-thickness defects of articular cartilage in rabbits. Osteoarthr Cartil.

[B41] Kalbhen DA, Blum U (1977). Hypothesis and experimental confirmation of a new pharmacological model of osteoarthrosis. Arzneimittelforschung.

